# Supplementary material for: Sleep restriction caused impaired emotional regulation without detectable brain activation changes—a functional magnetic resonance imaging study
Source: R Soc Open Sci. 2019 Mar 27;6(3):181704. doi: 10.1098/rsos.181704 (PMC6458356; doi:10.1098/rsos.181704)
Supplement: Supplemental material [file rsos181704supp1.docx]

**Sleep restriction caused impaired emotional regulation but no detectable brain activation changes – a functional magnetic resonance imaging study in younger and older adults**

**Supplemental materials**

Sandra Tamm^1,2^, Gustav Nilsonne^1,2^, Johanna Schwarz^2,3^, Armita Golkar^1,3^, Göran Kecklund^2^,

Predrag Petrovic^1^, Håkan Fischer^3^, Torbjörn Åkerstedt^1,2^, Mats Lekander^1,2^

Affiliations

1. Department of Clinical Neuroscience, Karolinska Institute

2. Stress Research Institute, Stockholm University

3. Department of Psychology, Stockholm University

**Method**


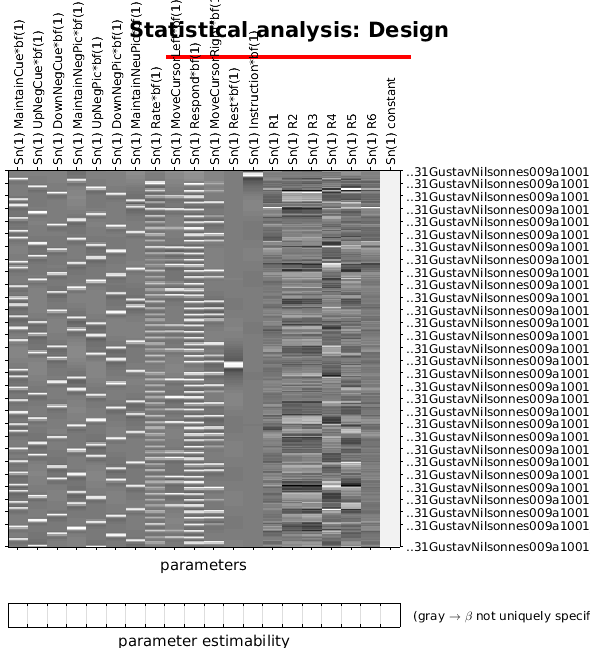

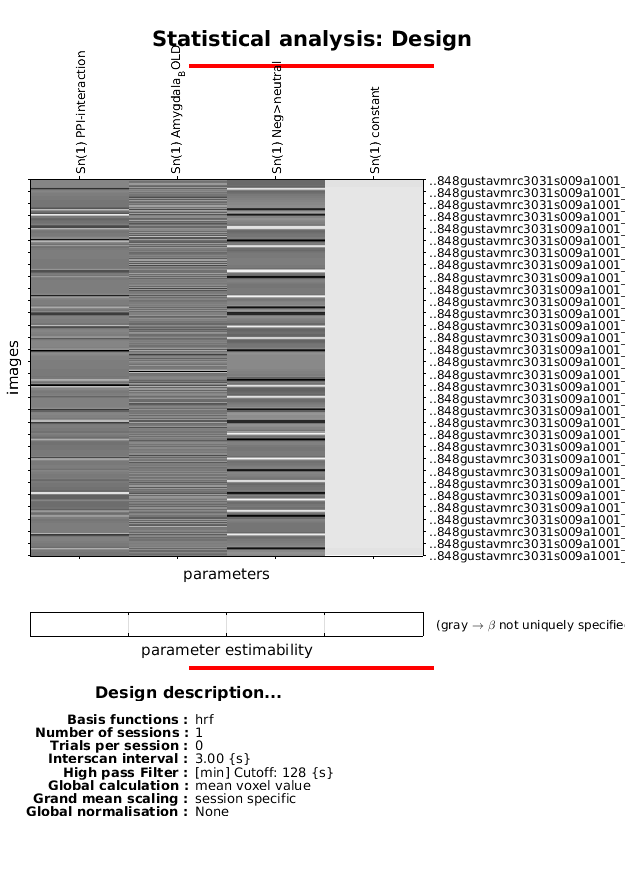


Supplemental figure 1. Design matrices for first level (left panel) and first level PPI analysis (right panel)

**Regions of interest**

For hypothesis testing, we used a region of interest (ROI) analysis with anatomical regions, as shown below. The dorsolateral prefrontal and lateral orbitofrontal ROIs were spherical ROIs based on the peak coordinates from the meta-analysis by Kalish et al, with a radius of 15 and 10 mm respectively. For the amygdala we used the whole anatomical region, defined based on the AAL in the WFU pickatlas.

Supplemental figure 2. Regions of interest for dorsolateral prefrontal cortex, lateral orbitofrontal cortex and amygdala

**Results**

**Maintain > downregulate**

Supplemental table 1. Maintain > downregulate

**
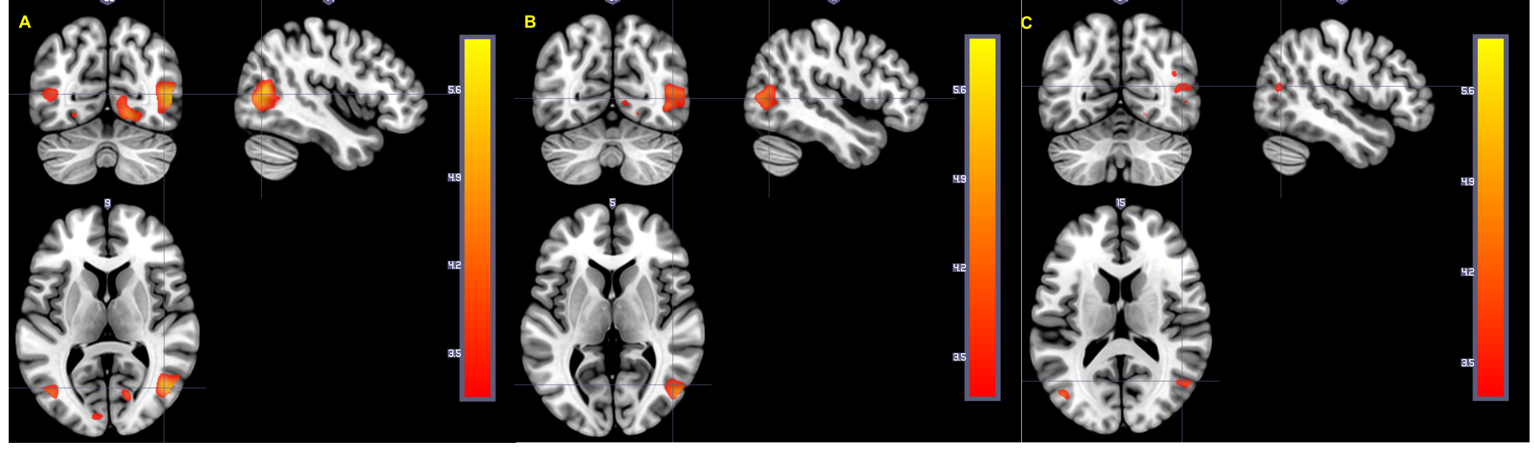
**

Supplemental figure 3. Maintain > downregulate in a) all b) young and c) old

**Effects of sleep restriction on ratings of success and unpleasantness (in older)**

In older participants, sleep restriction caused a general decrease in reported success as demonstrated by a main effect (-0.26 [-0.49, -0.04], *p* = 0.02, fig 6a). There was no sleep condition X stimulus type interaction (*p* = 0.43).

In older participants (n = 35), neither the main effect of sleep restriction (0.00 [-0.23, 0.23], p = 0.64, fig 6b) nor the sleep condition X valence interaction were significant (p = 0.80).

**Full factorial design**

As described in the main manuscript, older participants did not show the expected activity in dlPFC and lOFC when downregulating. Here we present the full factorial models (sleep*age), where age, sleep and the age*sleep interaction were investigated using linear contrast for the 1^st^ level contrasts negative > neutral, downregulate > maintain and upregulate > maintain.

**Age differences**

**Negative > neutral**


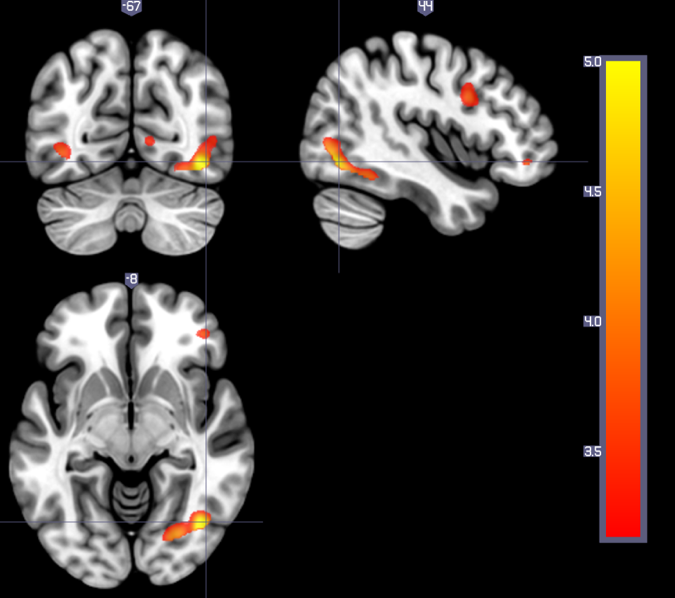


Supplemental figure 4. Young > old for negative > neutral


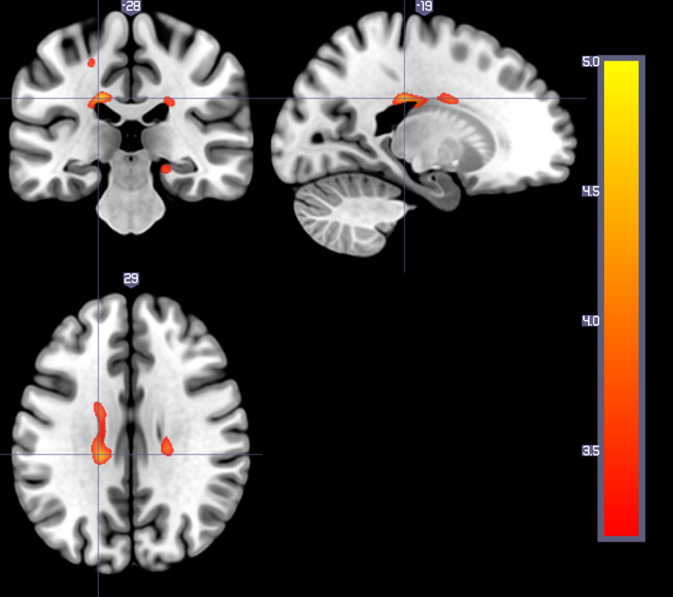


Supplemental figure 5. Old > young for negative > neutral

Supplemental table 2. Young > old for negative > neutral

Supplemental table 3. Old > young for negative > neutral

**Downregulate > maintain (negative)**


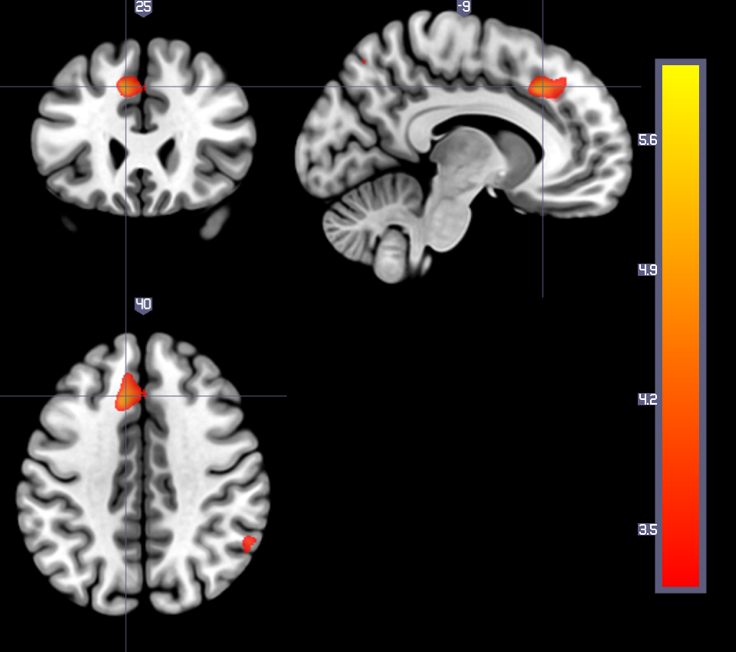


Supplemental figure 6. Young > old for downregulate > maintain


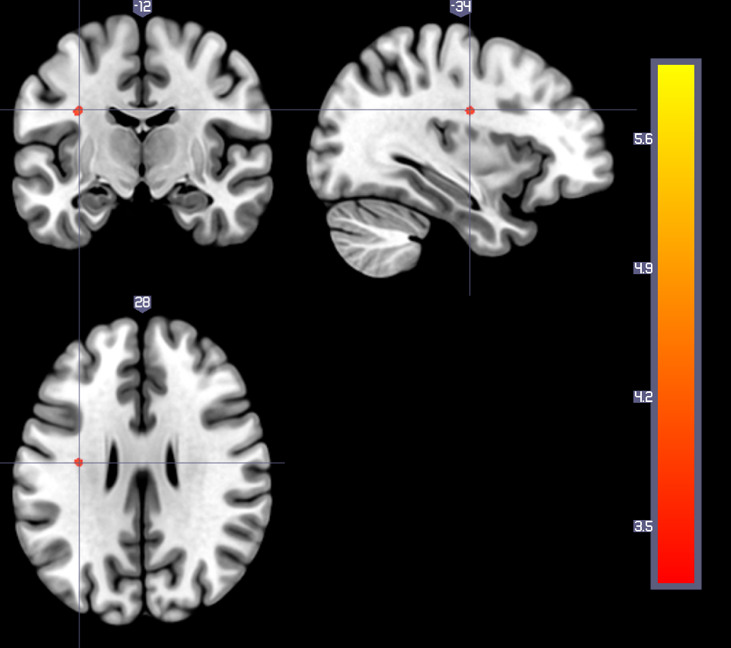


Supplemental figure 7. Old > young for downregulate > maintain

Supplemental table 4. Young > old for downregulate > maintain

Supplemental table 5. Old > young for downregulate > maintain

**Upregulate > maintain**

**
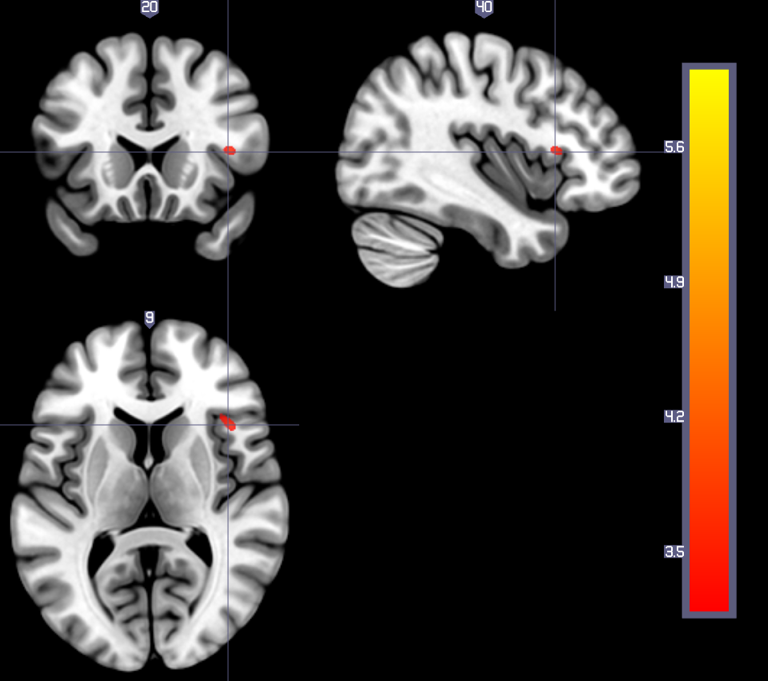
**

Supplemental figure 8. Young > old for upregulate > maintain


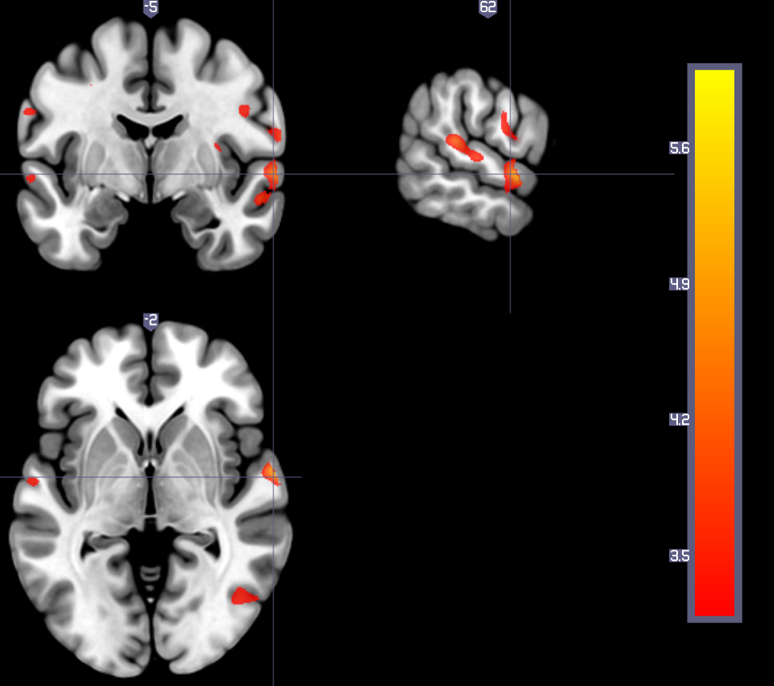


Supplemental figure 9. Old > young for upregulate > maintain

Supplemental table 6. Young > old for upregulate > maintain

Table 7. Old > young for upregulate > maintain

**Age*sleep interaction**

***Negative > neutral***

No voxels showed a significant effect for the age x sleep interaction on the contrast negative > neutral.

**Downregulate > maintain**

**
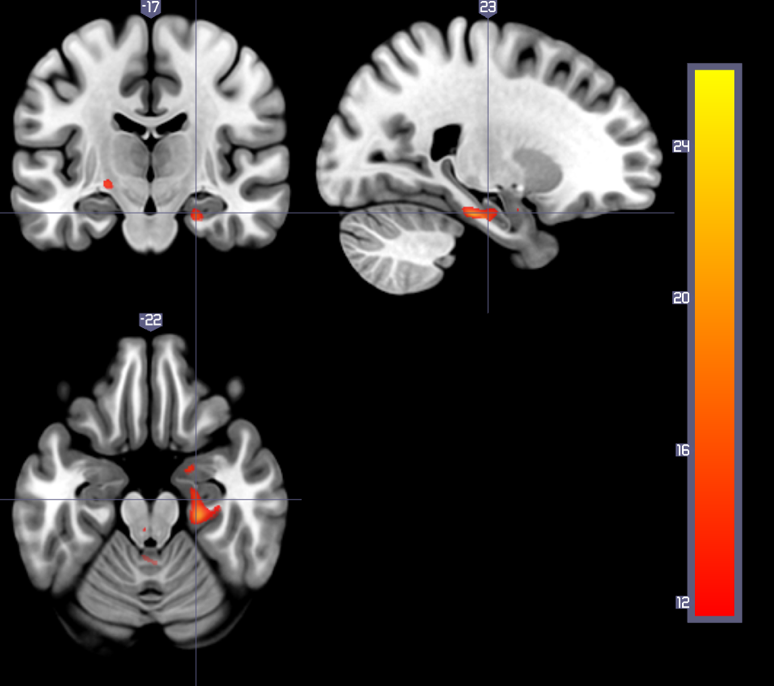
**

Supplemental figure 10. Age X sleep for downregulate > maintain

Supplemental table 8. Age X sleep for downregulate > maintain

**Upregulate > maintain**

**
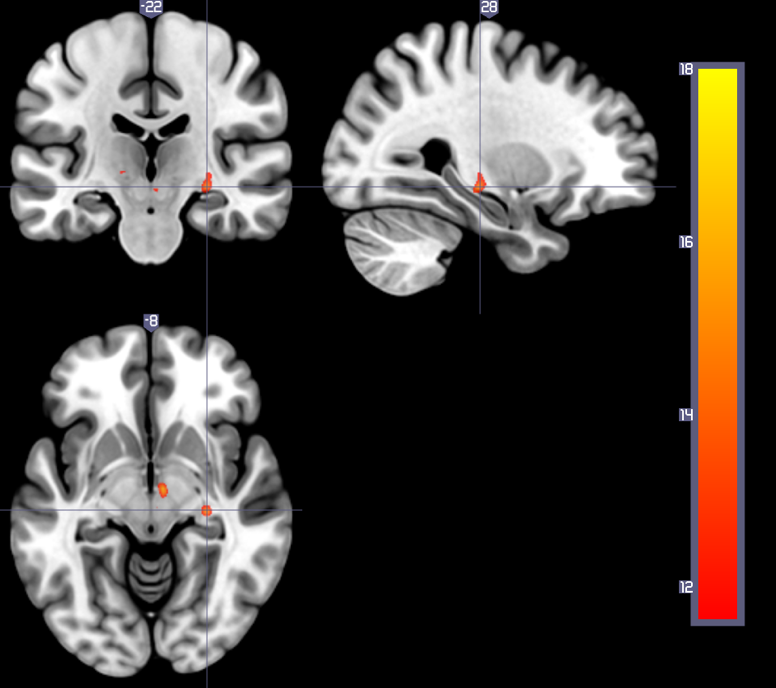
**

Supplemental figure 11. Age X sleep for upregulate > maintain

Supplemental table 9. Age X sleep for upregulate > maintain

**Effects of sleep restriction across the whole sample**

Since older participants did not show the expected effects associated with the task, the main analyses of effects of sleep restriction were performed on young. Here we present the effects of sleep restriction across the whole sample.

**Negative > neutral**

At whole brain level, no clusters showed a significant effect of sleep restriction. The effect of sleep restriction on amygdala activity for the contrast negative > neutral (ROI analysis) was not significant; left (-0.06 [-0.29, 0.17], p = 0.63), right (0.12 [-0.08, 0.32], p = 0.23).

**Downregulate > maintain**

Across age groups, one cluster in the orbitofrontal cortex was found, where more activity was observed in the full sleep condition compared to sleep restriction (suppl. table 10).

ROI analysis showed no significant effect of sleep restriction on amygdala; left (-0.08 [-0.32, 0.15], p = 0.48), right (-0.18 [-0.38, 0.01], p = 0.07). The effect of sleep restriction on dlPFC was not significant; left (-0.12 [-0.31, 0.07], p = 0.21), right (-0.15 [-0.40, 0.10], p = 0.25). Neither the effect of sleep restriction on lOFC was significant; left (-0.06 [-0.39, 0.26], p = 0.69), right (-0.25 [-0.53, 0.02), p = 0.07.


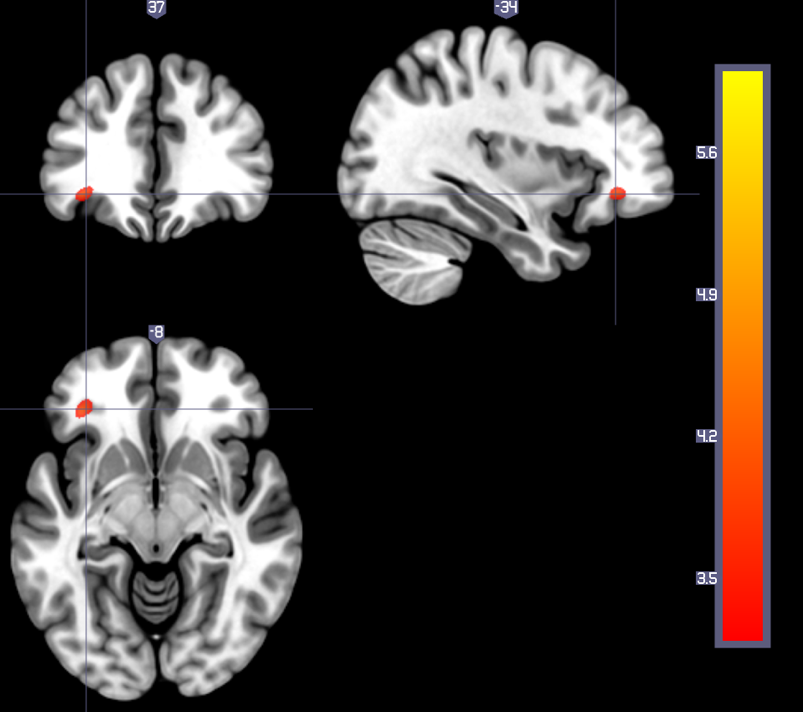


Supplemental figure 12. Full sleep > sleep restriction for downregulate > maintain

Supplemental table 10. Full sleep > sleep restriction on down > maintain

**Upregulate > maintain**

No voxels showed more activity in the full sleep condition compared to sleep restriction for the contrast upregulate > maintain.

**PPI, ROI-analysis**

Supplemental table 11. Effects of sleep restriction on connectivity from amygdala in young

Table 12. Supplemental table 11. Effects of sleep restriction on connectivity from amygdala in young

**Pupil diameter and heart rate**

Heart rate

Heart rate was normalised to the 4 seconds preceding the instruction (arrow) onset and plotted per stimulus type in fig 13. Stimulus onset caused a deceleration in heart rate for all stimulus types. To investigate the effect of stimulus type, sleep restriction and age group, heart rate index over the 5 seconds when the IAPS picture was shown was averaged for each event and entered into a mixed model. Compared to maintaining neutral, all stimulus types caused a significantly smaller deceleration; maintain negative (0.010 [0.001, 0.019], *p* = 0.02), downregulate (0.011 [0.003, 0.020], *p* = 0.01) and upregulate (0.009 [0.001, 0.019), *p* = 0.03). The type of instruction across negative pictures did not significantly change the response. Sleep restriction did not have any main effect on heart rate, nor did sleep restriction interact with stimulus type. Age did likewise not have any significant main effect or interaction with sleep restriction or stimulus type, see suppl. table 13 for details.


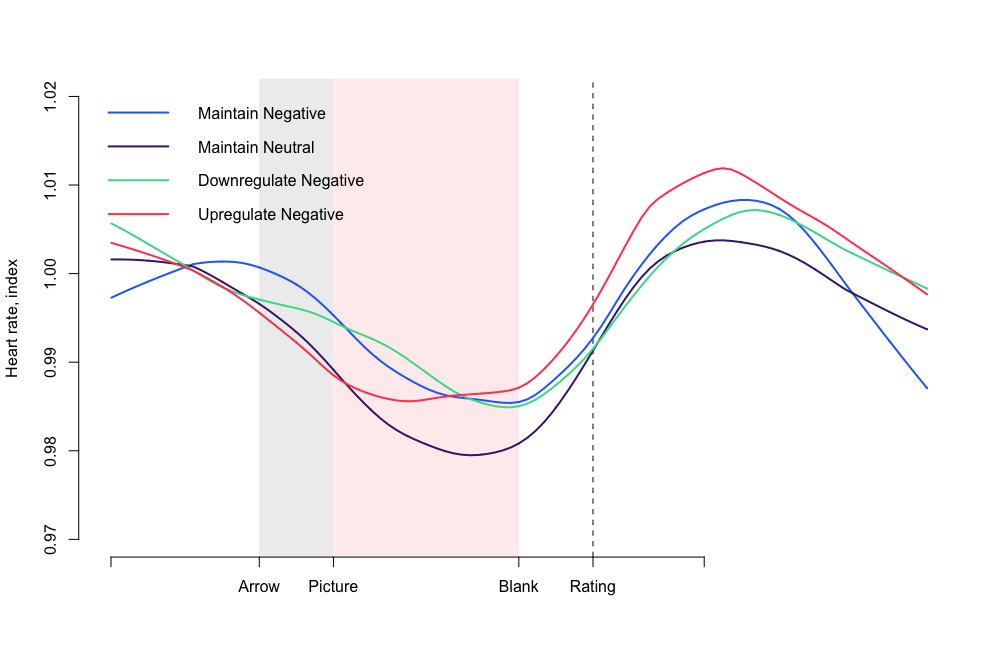


Supplemental figure 13. Heart rate responses to all stimulus types. Neither age nor sleep restriction was associated with any significant effects on heart rate responses

Pupil diameter

A mean time course per condition was inspected (fig 14), showing pupil constriction after onset of arrow. With 3639 events from 99 sessions in 73 participants, a mixed-effects model showed significantly less constriction for maintain negative compared to maintain neutral (*p* = 0.03) during the stimulus presentation. No other effects of stimulus type, sleep restriction or age was significant, see suppl. table 14.


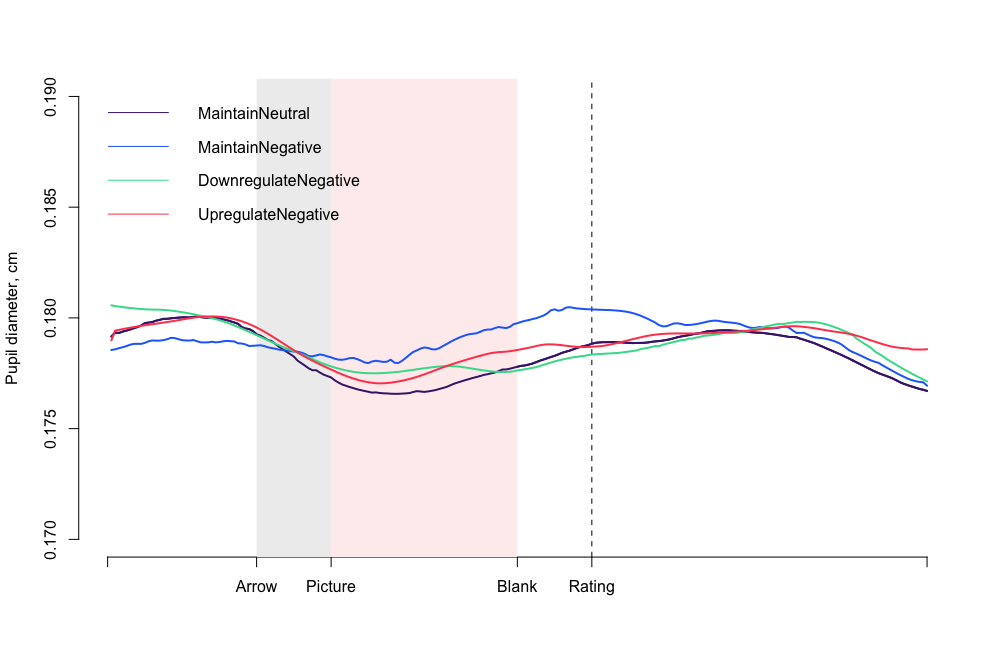


Figure 14. Pupil diameter responses. Less constriction was seen for maintain negative compared to maintain neutral. No other effects were significant.

Table 13. Full models of heart rate

Table 14. Full models of pupil diameter
